# Deciphering the Genetic Basis of Lodging Resistance in Wild Rice *Oryza longistaminata*

**DOI:** 10.3389/fpls.2020.00628

**Published:** 2020-05-29

**Authors:** Weixiong Long, Dong Dan, Zhengqing Yuan, Yunping Chen, Jie Jin, Weilong Yang, Zhihong Zhang, Nengwu Li, Shaoqing Li

**Affiliations:** State Key Laboratory of Hybrid Rice, Key Laboratory for Research and Utilization of Heterosis in Indica Rice of Ministry of Agriculture, Engineering Research Center for Plant Biotechnology and Germplasm Utilization of Ministry of Education, College of Life Science, Wuhan University, Wuhan, China

**Keywords:** lodging resistance, QTLs, *Oryza longistaminata*, wild rice, stem diameter

## Abstract

The abuse of fertilizer results in tall rice plants that are susceptible to lodging and reduced plant yield. Hence, it is important to identify and utilize the quantitative trait loci (QTLs)/genes for lodging resistance breeding. *Oryza longistaminata* exhibits a strong stem and high biomass productivity, which could be a candidate gene pool for cultivars lodging resistance improvement. Here, a set of 152 BC_2_F_20_ lines derived from a cross between a cultivated line 93-11 and *O. longistaminata* was evaluated for lodging resistance. QTL mapping analysis combined with single-nucleotide polymorphism (SNP) marker derived from high-throughput sequencing identified 12 QTLs for stem diameter (SD), 11 QTLs for stem length (SL), and 3 QTLs for breaking strength (BS). Of which, 14 QTLs were first identified from *O. longistaminata*. A major QTL, *qLR1*, which was delimited to a region ∼80 kb on chromosome 1, increased stem diameter, stem length, and breaking strength. Another major QTL, *qLR8*, that was delimited in an interval ∼120 kb on chromosome 8, significantly enhanced the breaking strength. These results provide evidence that *O. longistaminata* can be exploited to develop lodging-resistant rice lines.

## Introduction

Lodging in cereal crops is a major problem that results in decreased grain yield and deteriorated grain quality (Berry et al., 2004). Development of lodging-resistant varieties to cope with this challenge has been widely attended to increase yield in rice, maize, and other crops (Zhu et al., 2013; Peng et al., 2014; Yadav et al., 2017). Plant breeders have reduced lodging risk by introducing the semi-dwarf gene *sd1*, known as the “Green Revolution Gene” (Peng et al., 1999). However, recent studies showed that semi-dwarf trait in rice limits photosynthesis and biomass production leading to a yield penalty (Murai et al., 2002). Additionally, it may have a negative pleiotropic effect on culm morphology (Okuno et al., 2014). In other words, the gibberellin synthesis gene (*sd1*) widely applied in Green Revolution rice also reduces the culm strength by decreasing culm diameter, which makes it difficult to further improve lodging resistance by one semi-dwarf genes alone. Thus, it is important to search for alien genes favorable for breeding lodging-resistant rice.

Lodging resistance is a complex quantitative trait, which is affected by many factors, such as culm morphology, culm diameter and length, cellulose content, and environment conditions. Previous studies have shown that the culm diameter and size are highly correlated with the lodging resistance of rice (Kashiwagi and Ishimaru, 2004; Kashiwagi et al., 2006; Fan et al., 2018; Sowadan et al., 2018). Ookawa et al. (2010) identified four QTLs for bending moment at breaking and section modulus of the fourth internodes derived from two indica rice varieties with strong culms. Similarly, Kashiwagi et al. (2008) detected five QTLs for pushing resistance from a backcross between Nipponbare and Kasalath. Shailesh Yadav et al. (2017) mapped 12 QTLs for lodging resistance from a backcross between Swarna and Moroberekan. Moreover, three functional genes including *prl5*, *SCM2*, and *SCM3* have been identified to regulate stem diameter. Interestingly, *SCM2* increased not only culm strength but also spikelet number. However, few reports have been made about lodging resistance in wild rice apart from three QTLs for culm-base thickness derived from pLIA1, which carried *Oryza longistaminata*’s chromosome segments (Gichuhi et al., 2016). Hence, exploitation of new QTLs or genes from wild rice contributing to lodging resistance will help enrich the gene pool for improvement of lodging resistance in rice.

The perennial wild species *O. longistaminata*, which showed large stem diameter, thick stem wall, and high biomass production, is believed to help improve rice lodging resistance (Khush, 1997). In our study, we conducted QTL analysis of potential lodging resistance of *O. longistaminata* by evaluation of stem diameter, stem length, and breaking strength using an advanced backcross inbred line (BIL) population derived from a cross between *O. longistaminata* and 93–11. Totally, 26 QTLs for lodging resistance were detected, of which 12 QTLs for stem diameter (SD), 11 QTLs for stem length (SL), and three QTLs for breaking strength (BS) were derived from *O. longistaminata*. These novel QTLs will lay the foundation for breeding strong lodging resistant rice and broaden our understanding of the genetic basis of rice lodging resistance.

## Materials and Methods

### Plant Materials and Field Experiments

The recurrent indica rice variety 93–11, wild rice *O. longistaminata*, and 152 backcross inbred lines (BILs) derived from a cross between them were used in this research. Field experiments were conducted in Linshui, Hainan province during the rice-growing season from late October to late March and in Wuhan, Hubei province during mid-May to late-September in 2017 and 2018. Each BIL was planted in five rows with 10 plants in each row at a spacing of 20 cm × 16.5 cm. A randomized complete block design with three replications was used in each experiment.

### Measurement of Lodging Resistance-Related Traits

At 2 weeks after heading, five plants of each BIL and 93–11 were selected to investigate the lodging resistance traits. The stem diameter (SD) of the fifth internode from the top of each selected plant was measured using an electronic Vernier caliper in the field. Stem length of the internode (SL) from the top was measured from the ground to the base of the panicle. The breaking strength (BS), a parameter for the physical strength of the stem, was measured at the last internode of the plants using a plant lodging tester (YYD-1A, Zhejiang TOP Instrument Co., Ltd., China).

### Statistical Analysis

All the data were analyzed using SPSS 20.0 statistical software. Mean values of each trait were used for further QTL analysis. The standard deviation of the means was calculated using Microsoft Excel software. Correlations between the lodging resistance traits were evaluated using Pearson’s correlation.

### QTL Analysis and Sequence Analysis

A total of 2,432 bin markers was used to construct the genetic linkage map covering the whole genome as described by Jin et al. (2018). Inclusive composite interval mapping combined with additive mapping (ICIM-ADD) method was used to detect more precise lodging resistance QTLs (Meng et al., 2015). The significant logarithm of odds (LOD) value threshold for each trait was determined following the 5% permutation test with 1,000 replicates. The putative genes on the QTL region were identified based on the RIGW^1^.

## Results

### Phenotypic Evaluation of Lodging Resistance-Related Traits

Significant differences for the three measured traits were found between the two parents in both trials (Supplementary Figure 1 and Table 1). The wild rice performed better than 93–11 for all of the three lodging-related traits, indicating that *O. longistaminata* can efficiently improve the lodging resistance of rice.

**TABLE 1:** Performance of lodging associated trait in backcross inbred lines (BILs) within 2 years.

**Traits**	**Environment**	**Year**	**BILs**				
			**Min**	**Max**	**Average**	**SD**	**CV (%)**
Stem diameter (mm)	WH	2017	5.27	12.78	7.94	1.17	14.74
		2018	5.17	13.25	7.92	1.22	15.40
	HN	2017	5.79	12.23	8.66	1.17	13.51
		2018	5.52	13.00	8.68	1.24	14.29
Stem length (cm)	WH	2017	64.1	178.67	109.24	31.54	28.87
		2018	78.6	191.53	119.70	40.64	33.95
	HN	2017	41.08	157.90	83.59	29.54	35.34
		2018	49.11	181.67	103.02	30.62	29.72
Breaking strength (N)	HN	2017	5.81	29.68	14.95	4.64	31.04
		2018	6.13	30.28	15.12	4.65	30.75
	WH	2017	5.41	30.86	15.50	4.80	30.97
		2018	5.42	31.33	15.51	4.84	31.21

*WH and HN represent Wuhan and Hainan, respectively.*

**FIGURE 1:**
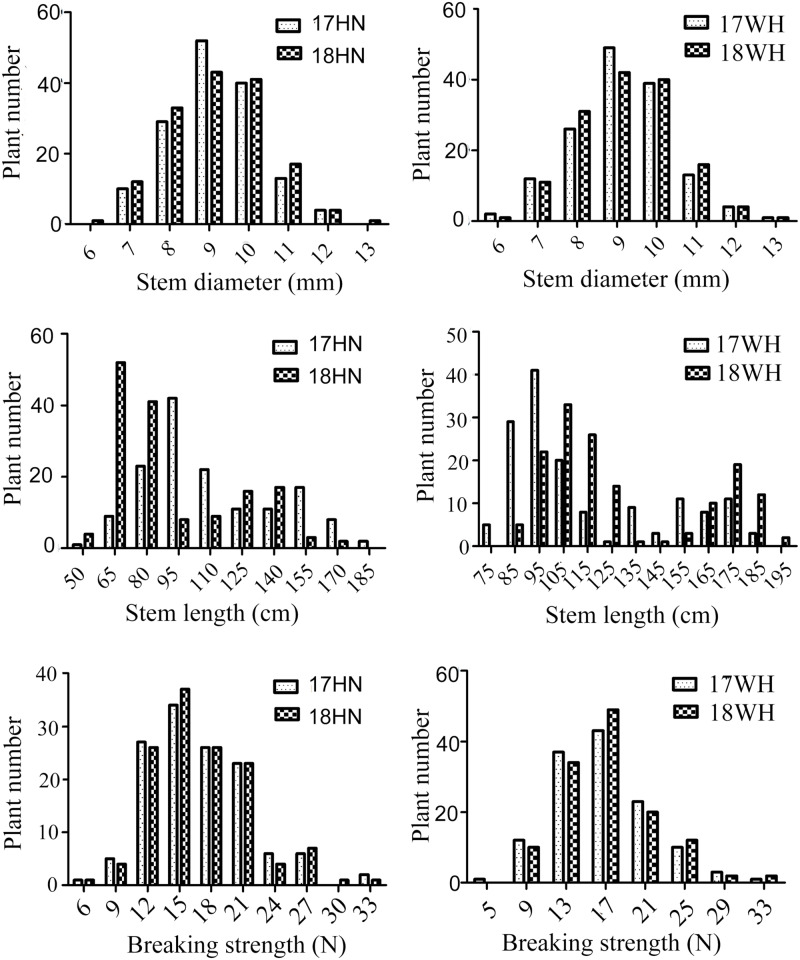
Phenotyping of lodging-resistant traits of 152 *Oryza longistaminata* backcross inbred lines (BILs). WH and HN present Wuhan and Hainan, respectively.

For the BIL population, the variation of stem diameter, stem length, and breaking strength in Hainan ranged from 5.79 to 12.23 mm, 41.08 to 157.90 cm, and 5.81 to 29.68 N, respectively, with an average of 8.66 mm, 83.59 cm, and 14.95 N, respectively, in 2017 (Figure 1). Correspondingly, the coefficient of variation (CV) of stem diameter (SD), stem length (SL), and breaking length (BS) was 13.51, 35.34, and 31.04%, respectively (Table 1). In 2018 at Hainan, the variation of SD, SL, and BS of *O. longistaminata* BIL population ranged from 5.52 to 13.00 mm, 49.11 to 181.67 cm, and 6.13 to 30.28 N, with an average of 8.68 mm, 103.03 cm, and 15.12 N, respectively. Correspondingly, the CV of SD, SL, and BS was 14.29, 29.72, and 30.75%, respectively.

In Wuhan, the SD ranged from 5.27 to 12.78 mm and 5.17 to 13.25 cm, with the CV of 14.73 and 15.40% in 2017 and 2018, respectively. The SL ranged from 64.1 to 178.67 cm, and 78.60 to 191.53 cm, with the CV of 28.87 and 33.95% in 2017 and 2018, respectively. The BS ranged from 5.41 to 30.86 N and 5.42 to 31.33 N, with the CV of 30.97 and 31.21% in 2017 and 2018, respectively (Table 1). These results indicate that the BS, SL, and SD of *O. longistaminata* BILs all showed great variation among different lines; BS and SL have more genetic diversity than SD regardless of environments.

### Correlations Among Lodging Resistance-Related Traits

Significant correlations (*P* < 0.05) were found between the traits studied (Supplementary Tables 2, 3). Breaking strength was found to be positively correlated with stem diameter and stem length in Hainan (*P* < 0.001). However, breaking strength was negatively correlated with stem length at Wuhan in 2018 (Supplementary Table 2). The negative correlation may be due to the photoperiod sensitivity of some BILs. These results indicate that wild rice *O. longistaminata* can improve the lodging resistance without the stem length reduction. In other words, it is possible to breed high yield and high biomass cultivars with high lodging resistance, which may provide appropriate genetic resources for breeding tall super yielding rice (Yuan, 2017).

### QTL Mapping of Lodging Resistance-Associated Traits

**FIGURE 2:**
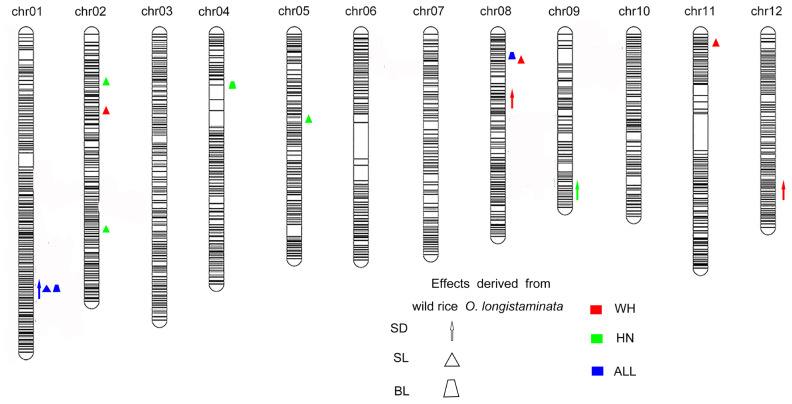
Quantitative trait loci (QTLs) for stem diameter, stem length, and breaking strength in *Oryza longistaminata* backcross inbred lines (BILs). The upward direction indicates the alleles of QTLs derived from *O. longistaminata* showing positive effects, and the center of the graphics indicates the QTLs region location. The red shapes show that the QTLs were detected in Wuhan, and the blue shapes represents the QTLs were identified in Hainan. The brown shapes mean QTLs mapped both two environments. Stem diameter (SD): arrow; stem length (SL): triangle; breaking strength (BS): trapezoid.

A total of 26 QTLs associated with stem diameter, stem length, and breaking strength were detected in the population of BILs (Figure 2). Among these, *O. longistaminata* supplied the superior allele at 14 QTLs (Tables 2, 3), while at the remaining 12 QTLs, the variety 93–11 supplied the superior alleles (Supplementary Tables 2, 3). Of which, eight QTLs including two for stem diameter, three for stem length, and three for breaking strength were identified from *O. longistaminata* in Hainan during the 2 years. Two QTLs, *qSD1.1* and *qSD9.1*, were detected for stem diameter. The QTL *qSD9.1* was detected in two consecutive years with a LOD of 3.17 and 4.99 and a PVE of 6.87 and 10.85% in 2017 and 2018, respectively. *qSD1.1* was identified in 2017, and it explained 13.68% of the phenotypic variation with a LOD of 5.78. The three QTLs (*qSL1.1*, *qSL2.1*, and *qSL2.2*) contributing to stem length were detected on chromosomes 1 and 2, and positive alleles were contributed by *O. longistaminata* (Figure 2). The QTL *qSL1.1* was detected in both 2017 and 2018, and it explained 59.40 and 67.03% of the phenotypic variation, respectively. QTLs *qSL2.1* and *qSL2.2* explained phenotypic variation of 5.53 and 10.22%, respectively. The three QTLs, *qBS1.1*, *qBS4.1*, and *qBS8.1*, contributing to stem breaking strength were mapped on chromosomes 1, 4, and 7. Among them, QTL *qBS1.1* was repeatedly detected across 2 years with the PVE of 5.07 and 1.86%, respectively. While *qBS4.1* and *qBS8.1* were detected only in 2017, they explained the phenotypic variance of 7.10 and 8.70%, respectively.

**TABLE 2:** Quantitative trait loci (QTLs) of lodging resistance derived from *Oryza longistaminata* in Hainan.

**Investigated traits**	**QTLs**	**Chr**	**Pos (cM)**	**L/Bin**	**R/Bin**	**L/bp**	**R/bp**	**LOD**		**PVE**		**Add**	
								**2017**	**2018**	**2017**	**2018**	**2017**	**2018**
Stem diameter	*qSD1.1*	1	226	1–171	1–172	35964905_36016970	35557085_35961004	5.78		13.68		0.49	
	*qSD9.1*	9	8	9–6	9–8	20987075_21077941	20967923_20984846	3.17	4.99	6.87	10.85	0.63	0.80
Stem length	*qSL1.1*	1	216	1–159	1–160	36587534_36864307	36569506_36587533	31.22	39.27	59.40	67.03	25.55	25.87
	*qSL2.1*	2	145	2–201	2–202	8863323_8895438	8841866_8863322	4.58		5.53		9.28	
	*qSL2.2*	2	119	2–247	2–148	18779567_18968062	18713595_18770186		3.77		10.22		2.57
Breaking strength	*qBS1.1*	1	216	1–161	1–162	36539238_36569505	36515725_36536296	4.87	5.07	13.70	16.76	1.86	2.06
	*qBS4.1*	4	120	4–97	4–98	19770490_20005045	19663208_19770489	2.66		7.10		2.14	
	*qBS8.1*	8	32	8–32	8–33	8075498_8118334	8118335_8196568	3.30		8.70		2.21	

**TABLE 3:** Quantitative trait loci (QTLs) associated with lodging resistance derived from *Oryza longistaminata* identified in a population of backcross inbred lines (BILs) evaluated in 2017 and 2018 in Wuhan and Hainan, China.

**Investigated traits**	**QTLs**	**Chr**	**Pos (cM)**	**L/Bin**	**R/Bin**	**L/bp**	**R/bp**	**LOD**		**PVE**		**Add**	
								**2017**	**2018**	**2017**	**2018**	**2017**	**2018**
Stem diameter	*qSD8.1*	8	74	8–59	8–60	9870144_9978376	20727323_21034035	3.43	4.55	18.83	8.03	1.12	1.13
	*qSD1.1*	1	216	1–161	1–162	36539238_36569505	36515725_36536296		13.29		25.37		0.65
	*qSD12.1*	12	156	12–168	12–169	22817923_22863684	22863685_22983852		51.39		34.80		1.99
Stem length	*qSL1.1*	1	216	1–160	1–161	36587534_36864307	36569506_36587533	2.77	53.00	7.21	79.89	9.11	31.64
	*qSL1.2*	1	286	1–310	1–311	23526094_23553497	23494864_23526093	3.43		9.12		10.51	
	*qSL2.3*	2	189	2–252	2–253	10475930_10505727	10520137_10562305	2.70		8.09		22.59	
	*qSL8.1*	8	78	8–59	8–60	9870144_9978376	20727323_21034035		3.32		4.42		18.61
	*qSL11.2*	11	290	11–128	11–129	1_370955	1355747_137440		2.50		5.01		23.36
Breaking strength	*qBS1.1*	1	216	1–161	1–162	36539238_36569505	36515725_36536296		4.02		11.22		1.75
	*qBS8.1*	8	32	8–31	8–32	8075498_8118334	8118335_8196568		3.78		10.22		2.57

A total of 10 QTLs for lodging-related traits were derived from *O. longistaminata* and were detected in Wuhan. Three QTLs (*qSD1.1*, *qSD8.1*, and *qSD12.1*) for stem diameter were detected on chromosomes 1, 8, and 12. The QTL *qSD8.1* was consistently detected in 2017 and 2018 with a LOD of 3.43 and 4.55, with the PVE of 18.33 and 8.03%, respectively. The QTLs *qSD1.1* and *qSD12.1* were identified in 2018 and explained phenotypic variation of 25.37 and 34.80%, respectively. Five QTLs, *qSL1.1*, *qSL1.2*, *qSL2.3*, *qSL8.1*, and *qSL11.2*, which were associated with stem length were mapped on chromosomes 1, 2, 3, 8, and 11, respectively. The QTL *qSL1.1* was detected in both years and explained phenotypic variation of 7.21 and 79.89%, respectively. The QTLs *qSL1.2* and *qSL2.3* were identified in 2017 and had PVE of 9.12 and 8.09%, respectively. QTLs *qSL8.1* and *qSL11.2* were mapped in 2018 with a LOD value of 3.32 and 2.50 and PVE of 4.42 and 5.01%, respectively. Two QTLs controlling breaking strength, *qBS1.1* and *qBS8.1*, were identified in 2018 with PVE of 11.22 and 10.22%, respectively.

### Colocalization and Stability of QTLs Associated With Lodging Resistance

To investigate the genetic effects of the QTLs responsible for lodging resistance, all the QTLs for which *O. longistaminata* had the superior allele at two sites were further analyzed. The QTLs *qSD1.1*, *qSL1.1*, and *qBS1.1* were colocalized on chromosome 1 (Figure 2). Another QTL, *qBS8.1*, was detected both in Wuhan and Hainan. The QTL hotspot *qSD1.1*/*qSL1.1*/*qBS1.1* explained 25.37% of the variance for stem diameter, 79.89% for stem length, and 16.76% for breaking strength, which indicates that this locus may play an important role for lodging resistance in rice. What is more, this pleiotropic QTL was first detected in wild rice, and we named it lodging resistance 1 (*qLR1*). Meanwhile, another QTL, *qBS8.1*, explained lodging resistance variance for breaking strength of 8.70 and 10.22% at Hainan and Wuhan, respectively (Tables 2, 3) and has been renamed as lodging resistance 8 (*qLR8*).

### Confirmation of the *qLR1* and *qLR8* for Lodging Resistance

**FIGURE 3:**
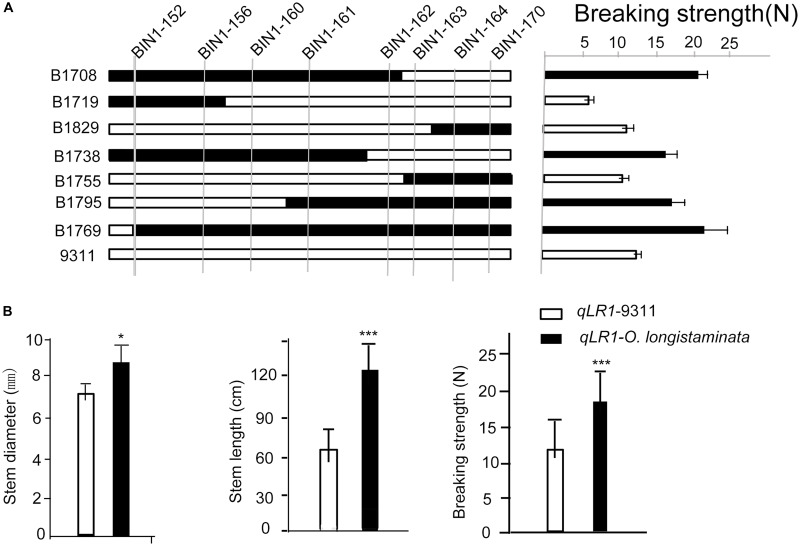
Confirmation of the quantitative trait loci (QTLs) of *qLR1*. **(A)** Verification of the *qLR1* using seven backcross inbred lines (BILs) to delimit it to an interval between molecular marker between Bin1-161 and Bin1-162. Black rectangle shows the homozygous derived from *Oryza longistaminata*, white rectangle indicates the homozygous from 93-11. **(B)**. *qLR1* effect value analysis of stem diameter, stem length, and breaking strength in *O. longistaminata* BILs. White shapes mean the alleles from parent 93-11; black shapes indicate the homozygous genotype shared by *O. longistaminata*.

To further confirm the function of the newly identified genetic locus of *qLR1* and *qLR8* in *O. longistaminata*, a high-resolution mapping with several BILs, including BIL 1708, 1719, 1829, 1738, 1755, 1795, and 1769, was performed. High-resolution mapping of several BILs possessing the superior *O. longistaminata* allele at *qLR1* helped narrow down the QTL to an interval between BIN1-161 and BIN1-162 (Figure 3A). Among the BILs, the average breaking strength of the lines with the superior *O. longistaminata* allele at *qLR1* was 19.60 N, which was significantly higher than those without *qLR1* (14.32 N, *P* < 0.001). Furthermore, the average stem diameter and stem length of the lines carrying *qLR1* was 9.72 mm and 123.12 cm, which were significantly (*P* < 0.01) larger than the lines without *qLR1*, which had an average stem diameter and stem length of 7.46 mm and 64.46 cm, respectively (Figure 3B). These results indicate that the *qLR1* from *O. longistaminata* can significantly increase lodging resistance.

**FIGURE 4:**
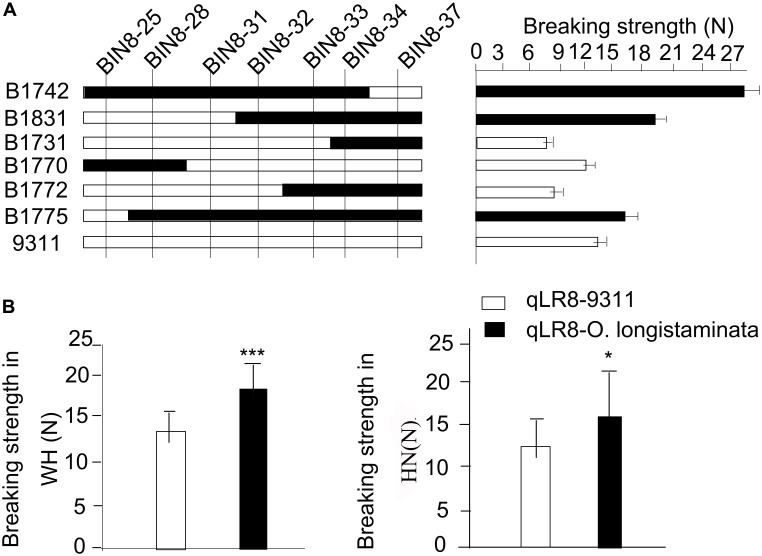
Validation of the quantitative trait loci (QTLs) of *qLR8*. **(A)** Verification of the *qLR8* using seven backcross inbred lines (BILs) to delimit it to an interval between two marker Bin8-32 and Bin8-33. **(B)** *qLR8* effect value analysis of breaking strength in both Wuhan and Hainan environments.

Similarly, after high-resolution mapping, we delimited the *qLR8* to both tightly linked markers BIN8-32 and BIN8-33 (Figure 4A). Then, we compared the genetic effects of *qLR8* on lodging resistance in the BILs. Results showed that the average breaking strength of BILs with *qLR8* was 18.89 N in Hainan, which was significantly higher than that of BILs, which lacked the superior *O. longistaminata* allele at *qLR8* with an average breaking strength of 14.34 N (Figure 4B). These results indicated that *qLR8* derived from *O. longistaminata* can significantly increase breaking strength.

To better understand if the two QTLs had additive effects, BIL 1704, 1720, 1728, 1730, 1742, 1779, and 1797-2, which carried *qLR1* and *qLR8*, were selected. The breaking strength of the seven lines ranged from 15.92 to 29.68 N, with an average of 22.55 N. This was significantly higher than the average value of the BILs carrying only *qLR1* (19.60 N) and the average value of the BILs carrying only qLR8 (18.89 N) (Supplementary Figure 2). These results indicate that two QTLs can significantly increase the lodging resistance of rice than either one QTL. In summary, *qLR1* and *qLR8* from *O. longistaminata* can significantly increase the lodging resistance of rice.

## Discussion

Lodging resistance is an important trait that is necessary for achieving high grain yield in rice production (Khush, 1997). In the past 60 years, short plant stature was the major target for improvement of lodging resistance, but many of the dwarfing genes are rarely applied to rice breeding due to deleterious effects on other agronomic traits such as low fertility and bold grains (Wu Z. et al., 2018). The “Green Evolution” gene *sd1* is still the only dwarfing source predominantly used to produce semi-dwarf varieties in rice. Recently, some studies have shown the possibility of improving rice yield by increasing plant biomass (Jiang et al., 2016). Increasing plant height is an effective and feasible way to increase biomass from a morphological viewpoint (Donald, 1968; Ying et al., 1998; Ma and Yuan, 2015). In this study, some tall *O. longistaminata* BILs also exhibited larger stem diameter, more breaking strength, and strong resistance to lodging (Figure 3), indicating that wild rice *O. longistaminata* is a novel genetic resource for breeding strong, lodging-resistant rice.

Although more than 24 QTLs for lodging resistance-associated traits had been reported earlier with three of them having been cloned in cultivars (Ookawa et al., 2016; Mulsanti et al., 2018; Sowadan et al., 2018), only two major QTLs for lodging resistance had been cloned (Xie et al., 2017; Wu Y. et al., 2018). Gichuhi et al. (2016) had identified three QTLs for culm-base thickness derived from *O. longistaminata*. None of them was overlapped with our results, which suggests that *O. longistaminata* contains great potential gene resources for cultivated rice lodging resistance improvement. In this study, we identified 12 new QTLs for lodging resistance with positive alleles derived from wild rice *O. longistaminata* for the first time. In addition, *qLR1*, a pleiotropic QTL responsible for stem length, stem diameter, and breaking strength, was narrowed to a small region ∼0.65 cM covering ∼80 kb physical distance on chromosome 1 (Figure 3A and Table 3). Corresponding to the MH63RS1 reference genome^2^, the region contains only 10 predicted genes: two hypothetical proteins and eight functional genes (Table 4). Two genes draw our attention according to their gene annotation. First is MH01t0727100-1 encoding a UDP-glucuronate:xylan alpha-glucuronosyltransferase 1, which can enhance mechanical strength of the stem (Gao et al., 2020). Epigenetic plays an increasing important role for plant breeding and selection of adaptive traits. Second is MH01t0727800-1 encoding a CHD3-type chromatin-remodeling factor PICKLE, which is highly associated with variability of growth and gene expression (Zhang et al., 2014). Meanwhile, *qLR8*, another QTL contributing to stem breaking strength, was localized in an interval of 1.34 cM covering ∼120 kb according on the MH63RS1 genome. This region contained 21 predicted genes: eight hypothetical proteins and 13 functional genes (Table 4). Interestingly, protein RALF-like 33 impacts on acidification and cell expansion during growth and development according to previous report (Murphy and De Smet, 2014). These information will provide insight into further gene cloning combined with the release of the gold *O. longistaminata*’s genome. Taken all the identified QTLs into consideration, no QTL for breaking strength was detected from 93-11 (Supplementary Figure 3), which indicates that wild rice *O. longistaminata* can significantly improve the lodging resistance of moderance rice. These QTLs identified in wild rice gave an insight into the genetic basis of lodging resistance.

**TABLE 4:** Putative genes at two quantitative trait loci (QTLs) regions for *qLR1* and *qLR8*, associated with lodging resistance in rice.

**QTLs**	**Gene ID**	**Start site (bp)**	**End site (bp)**	**Function**
*qLR1*	MH01t0727000-1	36523094	36523616	Hypothetical protein
	MH01t0727100-1	36528435	36534828	UDP-glucuronate:xylan alpha-glucuronosyltransferase 1
	MH01t0727200-1	36538440	36543325	Probable pectinesterase/pectinesterase inhibitor 51
	MH01t0727300-1	36543583	36548625	Hypothetical protein OsI_04663
	MH01t0727400-1	36549701	36550356	Hypothetical protein
	MH01t0727500-1	36550414	36551069	Cation/calcium exchanger 1
	MH01t0727600-1	36555859	36557636	Acyl-[acyl-carrier-protein] desaturase 1; chloroplastic
	MH01t0727700-1	36557901	36559958	Pentatricopeptide repeat-containing protein
	MH01t0727800-1	36560108	36568738	CHD3-type chromatin-remodeling factor PICKLE
	MH01t0727900-1	36569726	36571938	Putative clathrin assembly protein
*qLR8*	MH01t0165000-01	8081091	8086676	Bifunctional epoxide hydrolase 2
	MH01t0165100-01	8087305	8088199	Hypothetical protein
	MH01t0165200-01	8088985	8089633	Unknown protein
	MH01t0165400-01	8104092	8104277	Hypothetical protein
	MH01t0165300-01	8103887	8106234	Hypothetical protein
	MH01t0165500-01	8109136	8112092	Putative gypsy-type retrotransposon RIRE2
	MH01t0165600-01	8115542	8118452	Putative retrotransposon protein
	MH01t0165800-01	8122688	8124339	Hypothetical protein
	MH01t0166000-01	8125214	8125939	Hypothetical protein
	MH01t0166200-01	8129037	8129919	Hypothetical protein
	MH01t0166300-01	8131762	8132962	Hypothetical protein
	MH01t0166400-01	8134838	8137516	Disease resistance protein RGA2
	MH01t0166500-01	8140661	8144868	Dynein 8 kDa light chain; flagellar outer arm
	MH01t0166600-01	8146496	8146816	Indole-3-acetic acid-induced protein ARG2
	MH01t0166700-01	8148809	8149501	Antigen-like protein
	MH01t0166800-01	8150263	8152690	Putative AC transposase
	MH01t0166900-01	8160667	8169675	Zinc finger CCCH domain-containing protein 4
	MH01t0167000-01	8170873	8174334	Probable U6 snRNA-associated Sm-like protein LSm4
	MH01t0167100-01	8177528	8177923	Protein RALF-like 33
	MH01t0167200-01	8184849	8185401	18.6 kDa class III heat shock protein
	MH01t0167300-01	8185788	8187121	Flowering-promoting factor 1-like protein 1

In rice breeding, lodging resistance is one of the most essential characters required for breeding super high-yielding rice. Grain yield is the product of harvest index (HI) and biomass, and previous studies have shown that increasing plant height can increase biomass. Here, two newly identified QTLs, *qLR1* and *qLR8*, both improved stem breaking strength in rice. Furthermore, the former can also improve stem diameter and stem length. These two QTLs do not overlap with any previous reports. It is expected that, when these two QTLs are pyramided together, they can greatly improve lodging resistance in rice. Thus these two QTLs can serve as new resources for breeding superyielding rice varieties.

## Data Availability Statement

The raw data for this study can be found in the BioProject ID PRJNA615752 on NCBI. The URL is https://www.ncbi.nlm.nih.gov/sra/?term=PRJNA615752.

## Author Contributions

SL and WL conceived and planned the work. WL, DD, ZY, YC, and WY performed phenotypic screening. SL, NL, and JJ developed the population. WL analyzed the genotypic data. WL, ZZ, and SL drafted the manuscript.

## Conflict of Interest

The authors declare that the research was conducted in the absence of any commercial or financial relationships that could be construed as a potential conflict of interest.

## Footnotes

1 http://rice.hzau.edu.cn/rice/

2 http://rice.hzau.edu.cn/rice/
