# Supplementary material for: Deciphering the Genetic Basis of Lodging Resistance in Wild Rice Oryza longistaminata
Source: Front Plant Sci. 2020 May 29;11:628. doi: 10.3389/fpls.2020.00628 (PMC7274161; doi:10.3389/fpls.2020.00628)
Supplement: Supplementary file 1 [file Data_Sheet_1.docx]

­­­­­­­­­**Supplementary Figures and Tables**


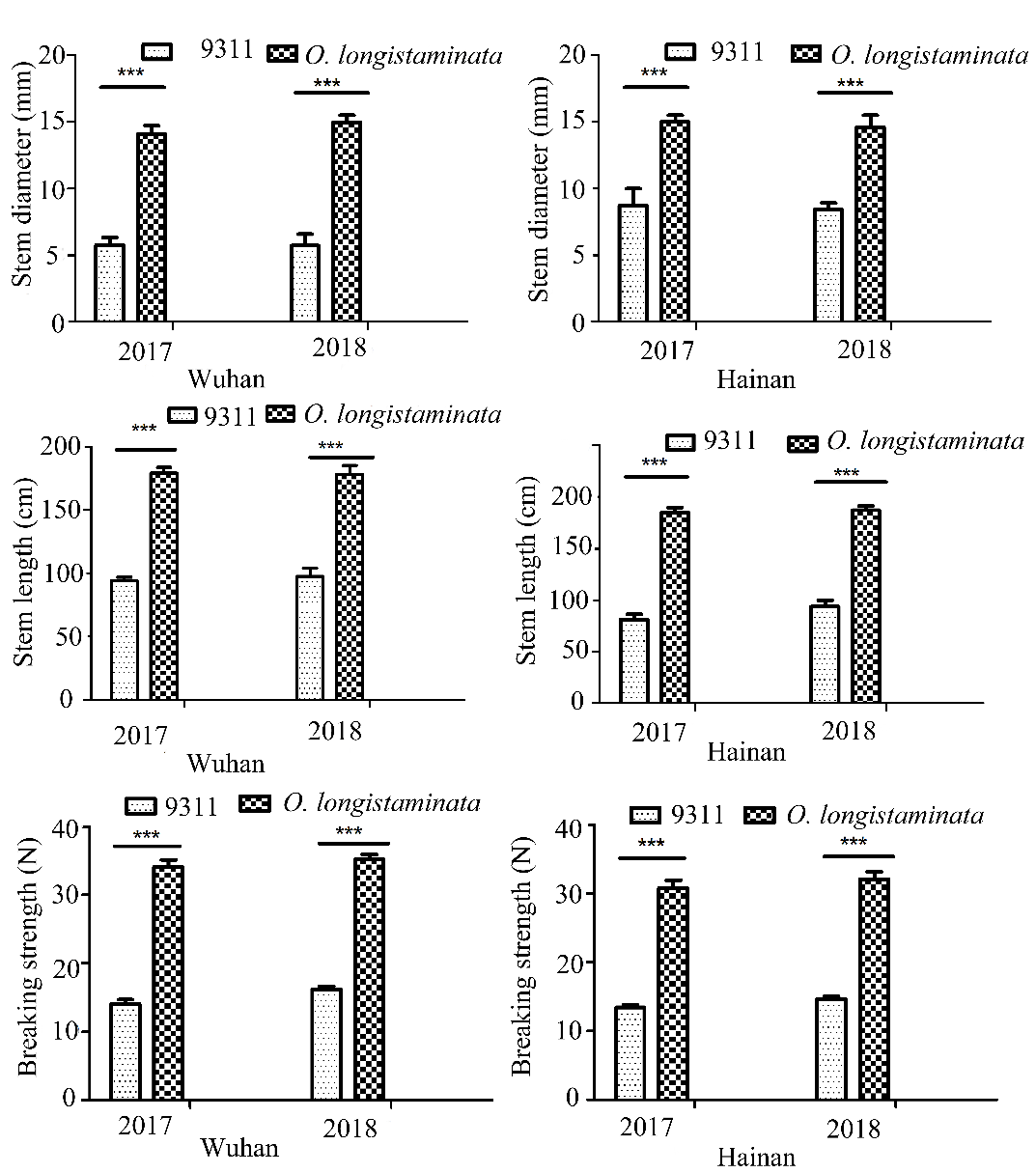


**Supplementary Fig. 1 Distribution frequency of the lodging-related trait of two parents 93-11 and O. longistaminata for two consecutive years at Wuhan and Hainan, China.** Left were identified at Wuhan and right was performed at Hainan.





**Supplementary Fig. 2 Pyramided QTLs *qLR* and *qLR8* significantly improve rice lodging resistance than carrying only one locus from wild rice *O. longistaminata***. Black rectangle stand as harbour two super alleles from wild rice exhait high breaking strength than carring only locus. P<0.05


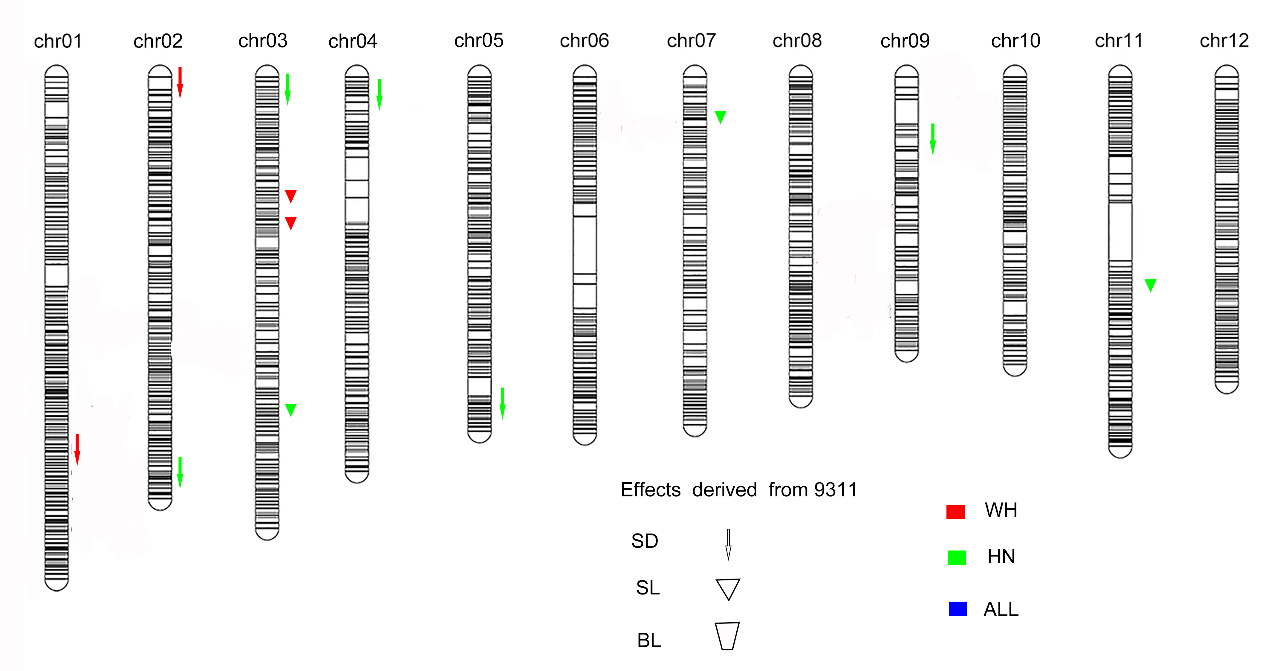


**Supplementary Fig. 3** **QTLs for seed vigor for artificial aging treatments detected in BIL population shared by 9311**. QTL for lodging related traits (stem diameter, stem length and breaking strength) derived from 93-11 were identified. Downward direction stands for the super alleles from 93-11. SD: arrow, SL: triangle, BS: trapezoid. Blue indicates identified at Wuhan, green stands for performed at Hainan, blue was identified at both site.

**Supplementary Table 1. Performance of lodging associated trait in BILs among two years**

| Traits | BIL | | | | |
| --- | --- | --- | --- | --- | --- |
|  | Min | Max | Average | SD | CV(%) |
| WH17SD(mm) | 5.27 | 12.78 | 7.94 | 1.17 | 14.74 |
| WH18SD(mm) | 5.17 | 13.25 | 7.92 | 1.22 | 15.40 |
| HN17SD(mm) | 5.79 | 12.23 | 8.66 | 1.17 | 13.51 |
| HN18SD(mm) | 5.52 | 13.00 | 8.68 | 1.24 | 14.29 |
| WH17SL(cm) | 64.1 | 178.67 | 109.24 | 31.54 | 28.87 |
| WH18SL(cm) | 78.60 | 191.53 | 119.70 | 40.64 | 33.95 |
| HN17SL(cm) | 41.08 | 157.90 | 83.59 | 29.54 | 35.34 |
| HN18SL(cm) | 49.11 | 181.67 | 103.02 | 30.62 | 29.72 |
| HN17BS(N) | 5.81 | 29.68 | 14.95 | 4.64 | 31.04 |
| HN18BS(N) | 6.13 | 30.28 | 15.12 | 4.65 | 30.75 |
| WH17BS(N) | 5.41 | 30.86 | 15.50 | 4.80 | 30.97 |
| WH18BS(N) | 5.42 | 31.33 | 15.51 | 4.84 | 31.21 |

**Supplementary Table 2. The correlation between the lodging resistance traits in Wuhan**

| Traits | Years | Stem diameter | | Stem length | | Break strength | |
| --- | --- | --- | --- | --- | --- | --- | --- |
|  |  | 2017 | 2018 | 2017 | 2018 | 2017 | 2018 |
| Stem diameter | 2017 | 1 |  |  |  |  |  |
|  | 2018 | 0.29*** | 1 |  |  |  |  |
| Stem length | 2017 | 0.24** | 0.39*** | 1 |  |  |  |
|  | 2018 | 0.41*** | 0.09 | -0.02 | 1 |  |  |
| Break strength | 2017 | 0.30** | 0.86*** | 0.38*** | 0.12 | 1 |  |
|  | 2018 | 0.24** | 0.38*** | 0.98*** | -0.03 | 0.38*** | 1 |

**Supplementary Table 3. The correlation among the traits in Hainan**

| Traits | Years | Stem diameter | | Stem length | | Break strength | |
| --- | --- | --- | --- | --- | --- | --- | --- |
|  |  | 2017 | 2018 | 2017 | 2018 | 2017 | 2018 |
| Stem diameter | 2017 | 1 |  |  |  |  |  |
|  | 2018 | 0.15 | 1 |  |  |  |  |
| Stem length | 2017 | 0.17 | 0.37*** | 1 |  |  |  |
|  | 2018 | 0.64*** | 0.18 | 0.18** | 1 |  |  |
| Break strength | 2017 | 0.18 | 0.93*** | 0.44*** | 0.22* | 1 |  |
|  | 2018 | 0.17 | 0.34*** | 0.94*** | 0.16** | 0.42*** | 1 |
